# The Genetic Architecture of Grain Yield in Spring Wheat Based on Genome-Wide Association Study

**DOI:** 10.3389/fgene.2021.728472

**Published:** 2021-11-15

**Authors:** Yuyao Li, Jingquan Tang, Wenlin Liu, Wenyi Yan, Yan Sun, Jingyu Che, Chao Tian, Hongji Zhang, Lihe Yu

**Affiliations:** ^1^Heilongjiang Bayi Agricultural University, Daqing, China; ^2^Heilongjiang Academy of Agricultural Sciences, Harbin, China; ^3^Crop Resources Institute, Heilongjiang Academy of Agricultural Sciences, Harbin, China; ^4^Keshan Branch, Heilongjiang Academy of Agricultural Sciences, Qiqihar, China

**Keywords:** GWAS, marker-assisted selection, single nucleotide polymorphism, *Triticum aestivum*, grain yield

## Abstract

Uncovering the genetic architecture for grain yield (GY)–related traits is important for wheat breeding. To detect stable loci for GY-related traits, a genome-wide association study (GWAS) was conducted in a diverse panel, which included 251 elite spring wheat accessions mainly from the Northeast of China. In total, 52,503 single nucleotide polymorphisms (SNPs) from the wheat 55 K SNP arrays were used. Thirty-eight loci for GY-related traits were detected and each explained 6.5–16.7% of the phenotypic variations among which 12 are at similar locations with the known genes or quantitative trait loci and 26 are likely to be new. Furthermore, six genes possibly involved in cell division, signal transduction, and plant development are candidate genes for GY-related traits. This study provides new insights into the genetic architecture of GY and the significantly associated SNPs and accessions with a larger number of favorable alleles could be used to further enhance GY in breeding.

## Background

Common wheat is the most important food crop worldwide and provides nearly 20% of the total caloric input to the global population. Grain yield (GY) improvement is one of the challenging goals in wheat breeding due to the complex genetic architecture and low heritability (He et al., 2010; Tester and Langridge, 2010; Gao et al., 2017; Wang et al., 2019). Heilongjiang and Jilin of China are the major spring wheat-producing regions, and yield potential in this region has been improved largely in the past decades (He et al., 2010; Zhou et al., 2014; Qin et al., 2015). However, the wheat production in this region is facing various threats, such as decreased groundwater, resulting in declining growing area, the irrigation frequency of wheat, and the bottleneck for yield potential of new cultivars through conventional breeding (He et al., 2010; Tester and Langridge, 2010).

Grain yield is a complex trait and influenced by many factors, particularly the genetic factors. GY-related traits included the spike number per unit area (SN), kernel number per spike (KNS), and thousand-kernel weight (TKW) (Ellis et al., 2005; Zhou et al., 2007; Rasheed et al., 2016; Gao et al., 2017; Li et al., 2018). Marker-assisted selection (MAS) is an effective tool for the further improvement of yield potential. Also, MAS is a key technique to increase the yield of wheat (He et al., 2010; Rasheed et al., 2016).

The effectiveness and reliability of MAS depends on the number of available genes and tightly linked markers for target traits. Until now, more than 70 genes have been cloned in wheat, among which 40 are associated with GY and related traits (Cui et al., 2014; Rasheed et al., 2016; Nadolska-Orczyk et al., 2017; Li et al., 2018; Wang et al., 2019). For all the cloned genes, about 150 functional markers or kompetitive allele-specific PCR (KASP) were developed (Rasheed et al., 2016). Besides this, more than 100 loci identified by genome-wide association study (GWAS) or biparental linkage mapping for GY-related traits are reported (Cui et al., 2014; Gao et al., 2017; Würschum et al., 2017; Li et al., 2018). However, identifying the novel genes or loci for GY is still important for wheat production.

Single nucleotide polymorphisms (SNP) provide an effective way to identify candidate genes for various traits (Zhu et al., 2008; Wang et al., 2014; Rasheed et al., 2016). Recently, the wheat 55, 90, and 660 K SNP arrays are gradually replacing simple sequence repeats (SSR) and diversity array technology (DArT) markers in genetic analysis in yield, disease resistance, end-use quality, and biotic or abiotic stress tolerance–related traits (Jin et al., 2016; Li et al., 2016; Liu et al., 2016, 2017; Valluru et al., 2017; Quan et al., 2021). Linkage analysis based on biparental populations and association mapping based on natural populations are two main ways to uncover the genetic analysis of complex traits (Zhu et al., 2008; Liu et al., 2017). Compared with linkage analysis, association mapping is based on linkage disequilibrium (LD) and offers an effective and reliable approach to uncover the genetic architecture of complex traits (Zhu et al., 2008; Liu et al., 2017). GWAS uses the natural germplasms, including wild types, landraces, released cultivars, and improved accessions, as materials and bypasses the time of developing biparental populations (Sela et al., 2014; Shi et al., 2017). Furthermore, traditional linkage analysis focuses on specific traits, whereas association mapping can be used to analyze various traits based on the same genotype data (Zhu et al., 2008). Nowadays, GWAS is commonly applied in genetic analysis of complex traits in wheat, such as GY-related traits, disease-related traits (stripe rust, leaf rust, or powdery mildew), and biotic and abiotic stress (Cui et al., 2011, 2014; Azadi et al., 2015; Beyer et al., 2019; Quan et al., 2021).

In China, Heilongjiang and Jilin are the main zones for spring wheat. In this study, 251 spring wheat accessions mainly originating from Heilongjiang and Jilin (1930–2020s) were selected. The aims of this study were to (1) detect the loci for GY and related traits in spring wheat, and (2) search for candidate genes for GY-related traits for further study.

## Materials and Methods

### Plant Materials and Field Trials

The diverse panel used in the present study contained 251 varieties mainly from Heilongjiang or Jilin province of China (Supplementary Table 1). The diverse panel was grown at Haerbin and Keshan in Heilongjiang province during the 2019 and 2020 cropping seasons. A randomized complete block design with three replicates was employed in field trials. Each plot comprised four 2.0-m rows spaced 20 cm apart with 40 seeds in each row. Agronomic management was performed according to local practices at each location.

### Phenotyping and Statistical Analysis

Six phenotypic traits related to GY were evaluated in all four environments, including the GY, spike number per unit area (SNU), SN, spike length (SL), KNS, and TKW. The middle two lines of plants were harvested in each plot at physiological maturity, and GY was measured after the seed water content dried to 14% and was expressed as kg ha^–1^. The investigation of the other five traits and statistical analysis were according to Li et al. (2018). BLUP estimation for six traits among four environments was calculated using the MIXED procedure (PROCMIXED) in SAS v9.3 (SAS Institute)^1^ following the formula:


y=Xb+Zu+e.

Of these, *y* is the observed phenotype, Xb is the fixed effects (environment), Zu is the random effect (genotype), and e is the residual effect.

### Genotyping, Population Structure, and Linkage Disequilibrium

The 251 accessions were genotyped using the wheat 55 K SNP arrays. SNP markers with missing data > 20% and minor allele frequency (MAF) < 0.05 were removed to avoid spurious alleles. The physical position for subsequent GWAS analysis followed the IWGSC RefSeq v2.1^2^.

Polymorphic and evenly distributed markers (one marker/LD block) were used to conduct population structure analysis. In this study, after filtering by MAF and missing data, 3000 polymorphic SNP markers (about 5 Mb for the whole genome of common wheat according to previous studies) evenly distributed on 21 chromosomes were analyzed in Structure v2.3.4 (Pritchard et al., 2000)^3^.

To verify the result, PCA and neighbor-jointing (NJ) trees were also estimated using the software Tassel v5.0 (Breseghello and Sorrells, 2006), respectively. After filtering using the Tassel v5.0, 5000 evenly distributed SNP markers were chosen to calculate LD for whole genomes using the full matrix and sliding window options in Tassel v5.0 (Breseghello and Sorrells, 2006). The details for population structure and LD decay analysis followed Liu et al. (2017). Variance components were used to calculate broad sense heritability (*h*_*b*_^2^) of GY-related traits as *h*_*b*_^2^ = σ*_*g*_*^2^/(σ*_*g*_*^2^ + σ*_*ge*_*^2^/r + σ_ε_^2^/re), where σ*_*g*_*^2^, σ*_*ge*_*^2^, and σ_ε_^2^ represent the genotype, genotype × environment interaction, and residual error variances, respectively, and e and r are the numbers of environments and replicates per environment, respectively.

### Genome-Wide Association Mapping

To control background variation and eliminate the spurious marker-trait associations (MTAs), the mixed linear model (MLM, PCA + K model) in Tassel v5.0 was used in consideration of the kinship matrix and population structure as follows:


y=μ+xβ+u+e.

Of these, y is the vector of observed phenotype, μ is the mean, x is the genotype, β is the effect of the SNP, u is the random effects due to genetic relatedness with Var (u) = σ^2^g K and Var (e) = σ^2^e, and K is the kinship matrix across all genotypes. The kinship matrix was treated as a random-effect factor and calculated by the software Tassel v5.0, whereas the PCA was considered as a fixed-effect factor and inferred by the Tassel V5.0 in MLM analysis. Due to Bonferroni–Holm correction for multiple testing (α = 0.05) being too conserved, and no significant MTAs were detected with this criterion, markers with an adjusted -log10 (*P*-value) ≥ 3.0 were regarded as significant markers. Manhattan plots and Q-Q plots were drawn based on R language (R 3.6.5).

### The Identification of Candidate Genes

Loci existing in two or more environments are considered stable. To identify candidate genes, the flanking sequences corresponding to the SNP markers (including the SNPs located in the LD decay interval for peak markers) significantly associated with GY-related traits are used in BLASTn and BLASTx searches against NCBI^4^ and ENA databases. Also, the annotation information for IWGSC 2.1 was also used to identify candidate genes.

## Results

### Phenotypic Evaluation

Significant and continuous variations of GY and related traits were observed in the diverse panel (Supplementary Table 1 and Supplementary Figure 1). The mean values of SNU, SN, SL, KNS, TKW, and GY were 131.1 (88.7–177.9), 16.6 (12.9–20.7), 10.6 (6.8–13.8), 36.0 (23.1–50.6), 32.4 g (20.5–41.2 g), and 4623.5 kg.hm^–1^ (2233.0–6987.4 kg.hm^–1^). ANOVA showed highly significant effects (*P* < 0.01) of genotypes, environments, and genotype × environment interactions on all traits (Table 1) that were exhibited. The broad sense heritability (*h*^2^) for SNU, SN, SL, KNS, TKW, and GY were 0.64, 0.52, 0.65, 0.71, 0.63, and 0.49, respectively, which indicates most of these traits are stable and mainly determined by genetic factors.

**TABLE 1:** ANOVA analysis for GY-related traits in 251 spring wheat accessions.

		*F*-value					
Source of variation	df	SNU	SN	KNS	SL	TKW	GY
Genotypes	250	120.4**	24.4**	94.4**	29.2**	85.8**	108.5**
Environments	3	380.9**	98.5**	302.1**	115.6**	258.5**	325.4**
Replicates (nested in environments)	2	18.2**	5.3**	13.2**	6.8**	13.6**	21.2**
Genotypes*Environments	749	9.2**	4.3**	5.6**	4.8**	6.3**	12.2**
Error	1425						

* and ** indicate significant at 0.05 and 0.01 level.

In this study, GY showed significant (*P* < 0.01) and positive correlations with SNU, SN, SL, KNS, and TKW (*r* ranged from 0.18 to 0.75). SNU showed significant (*P* < 0.01) and positive correlations with GY (*r* = 0.18), whereas significant and negative correlations with SN, SL, KNS, and TKW (*r* ranged from 0.19 to 0.24); SN showed significant (*P* < 0.01) and positive correlations with SL, KNS, and TKW (*r* ranged from 0.10 to 0.82); SL exhibited significant (*P* < 0.01) and positive correlations with KNS and TKW (*r* ranged from 0.21 to 0.71) (Supplementary Table 2).

### Marker Coverage and Genetic Diversity of the Physical Map

After filtering unqualified markers, 52,503 polymorphic SNPs were employed for construction of physical map and GWAS analysis (Supplementary Table 3 and Supplementary Figure 2). Among the polymorphic SNP markers, 34.9, 35.6, and 29.6% were from the A, B, and D genomes, respectively, indicating that the D genome has the lowest polymorphism (Supplementary Table 3). Of these, the chromosome 6B had more SNPs at 2691, whereas the 4D possessed only 1118 SNPs. The total length of the physical map is 14,058.8 Mb with an average marker density of 0.273 Mb per marker.

### Population Structure and Linkage Disequilibrium

**FIGURE 1:**
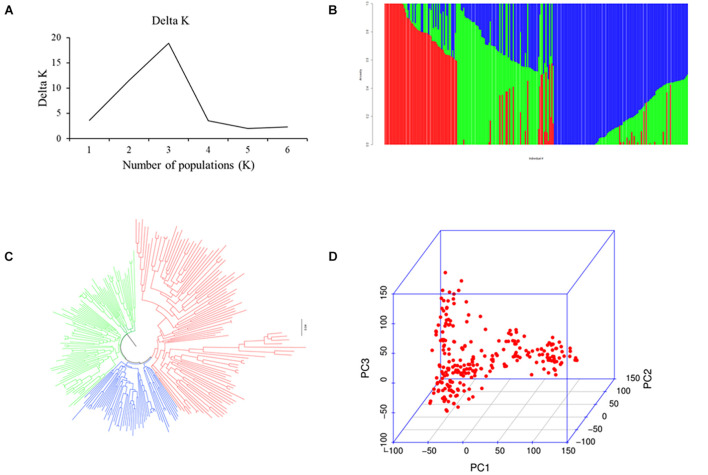
Population analysis for the 251 spring wheat accessions. **(A)** Delta K for structure analysis. **(B)** Population structure analysis. **(C)** Neighbor-joining (NJ) tree. **(D)** Principal components analysis (PCA) plots.

Population analysis indicates that all 251 wheat accessions were divided into three subgroups: subgroups I, II, and III. Of these, subgroup I contained 126 varieties mainly from Heilongjiang province ranging from the 1950s to 1980s, such as Kehong, Kehan 8, Kejian, and Xinshuguang 5; subgroup II had 75 varieties mainly from Heilongjiang province ranging from the 1990s to 2010s, such as Kechun 2, Kechun 9, Longmai 2, and Longmai 8; and subgroup III comprised 50 varieties mainly from Jilin province of China and foreign counties (United States, Canada, and Japan) (Figure 1). Furthermore, the average LD for the whole genome was 8 Mb (Supplementary Figure 3).

### GWAS

**FIGURE 2:**
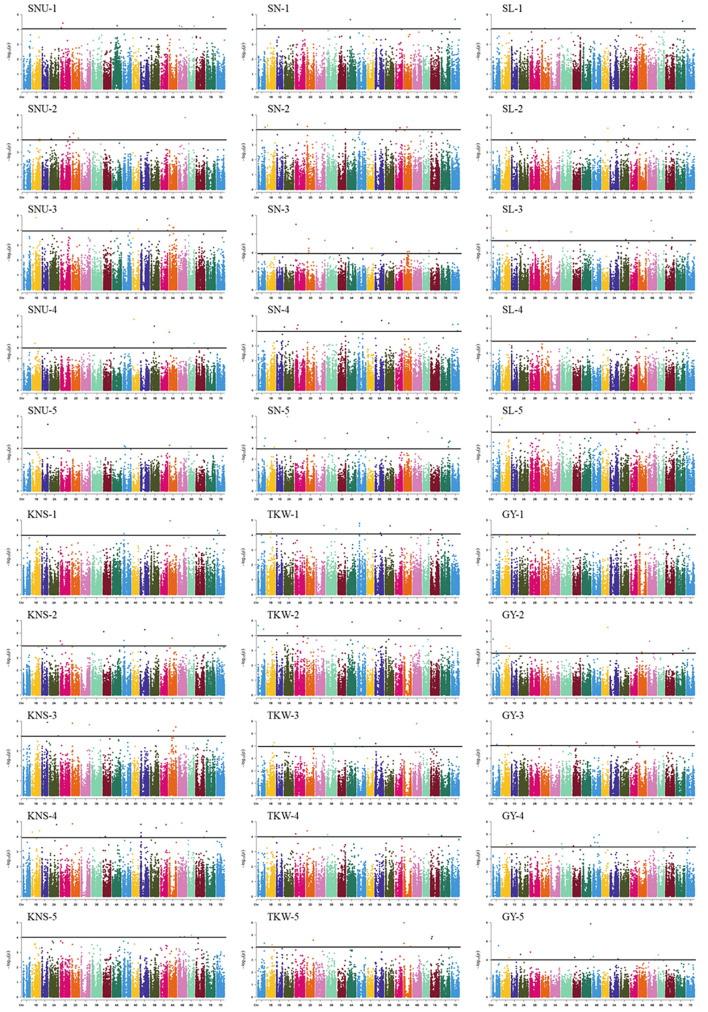
Manhattan plot for GY-related traits in 251 wheat accessions analyzed by the MLM in Tassel v5.0. SNU, spike number per unit area; SN, spikelet number; SL, spike length; KNS, kernel number per spike; TKW, thousand-kernel weight; GY, grain yield. The 1, 2, 3, 4, and 5 indicated the Haerbin, 2018, 2019; Keshan, 2018, 2019, and the best linear unbiased prediction (BLUP).

In total, 38 loci (162 MTAs) were identified associated with GY-related traits in this study (Table 2, Figure 2, Supplementary Table 4, and Supplementary Figure 4). Among these, 6 (1D, 3D, 5A, 6A, 6D, and 7B), 7 (2D, 3A, 3B, 5A, and 6A), 21 (1A, 1D, 2D, 3A, 3B, 3D, 4A, 4B, 4D, 5A, 5B, 6A, 6B, and 6D), 7 (1B, 2B, 5A, 6B, and 7D), 5 (1A, 4A, 4B, 1A, 4A, 1A, and 4A), and 12 (2B, 3A, 3D, 5D, 6A, 6D, 3D, 4A, 1D, and 2A) loci were detected for SNU, SN, SL, KNS, TKW, and GY, respectively and each explaining 7.7–9.3% of the phenotypic variances. Of these loci, two on chromosome 3D (22.8 Mb) and 6D (482.3–482.6 Mb) showed pleiotropic effects on SNU and GY; a locus on chromosome 5A (517.5–529.3 Mb) controlled both the SL and SNU; four loci on chromosome 2D (23.2–37.9 Mb), 3A (10.9–21.9 and 595.6 Mb), and 5A (20.7 Mb) had pleiotropic effects on SL and SN. Besides this, five loci on chromosome 1A (28.6–30.7, 307.1–321.1, and 338.9–346.0 Mb), 4A (653.4–687.1 Mb), and 4B (15.8–17.3 Mb) had pleiotropic effects on SL and TKW; two loci on chromosome 1D (252.4–284.1 Mb) and 3D (600.0–600.4 Mb) had significant effects on GY and KNS; and a locus on chromosome 2B (592.8 Mb) controlled both the KNS and GY. Another locus on chromosome 6A (2.5–4.0 Mb) had pleiotropic effects on GY, SN, and SL, whereas the loci of 6B (132.4–133.0 Mb), showed pleiotropic effects on SL and KNS.

**TABLE 2:** Loci for GY-related traits in 251 spring wheat accessions by association analysis.

Peak Marker^a^	Chromosome	Position (bp)^b^	*P*-value	*R*^2^ (%)	Interval (Mb)	Trait^c^	Favorable allele	References
*AX-110180733*	1A	28676837	4.5E-07	13.5	28.6–30.7	TKW/SL	C	Wang et al. (2011) and Li et al. (2019)
*AX-108817901*	1A	309183870	1.4E-05	9.3	307.1–321.1	TKW/SL	C	
*AX-109971512*	1A	338900660	5.2E-05	8.5	338.9–346.0	SL	C	
*AX-108850659*	1A	370774742	1.4E-05	9.3	370.7–370.8	SL	C	
*AX-110363533*	1A	587798859	1.8E-05	9.2	587.8–594.7	SN	G	
*AX-110953049*	1B	103157084	1.7E-05	9.1	100.8–103.2	YM/NS	G	
*AX-86175573*	1D	284057669	6.1E-05	8.2	252.4–284.1	YM/NS	C	
*AX-94767476*	1D	430118481	6.3E-06	10.1	430.1–430.3	SL	C	
*AX-111041231*	2A	34739520	2.3E-05	9.0	34.7	YM	A	Li et al. (2019)
*AX-111470278*	2B	592759916	3.8E-06	10.4	592.8	SN/YM	C	
*AX-86163393*	2D	37927966	1.9E-05	9.0	23.9–37.9	SL/NSS	C	
*AX-110090611*	2D	556224959	1.6E-06	11.5	556.2–556.3	SL	C	
*AX-111707600*	3A	10841219	3.1E-05	11.7	10.9–21.9	YM/NSS	G	Li et al. (2019)
*AX-110922897*	3A	596035891	3.4E-06	10.5	596.0	NSS/SL	G	
*AX-109490522*	3A	696351940	8.0E-06	9.8	696.4–700.9	SL	A	Azadi et al. (2015) and Li et al. (2019)
*AX-109379472*	3B	481361479	3.6E-05	8.8	480.4–481.4	NSS	G	
*AX-108791993*	3B	589834517	3.0E-05	8.8	589.9	SL	A	
*AX-94879852*	3D	22763685	6.4E-05	8.2	22.8	NS/YM	A	
*AX-109181055*	3D	600142463	1.6E-06	11.4	600.0–600.4	NS/YM	A	Li et al. (2019)
*AX-111662342*	4A	640213293	4.6E-05	8.0	640.2	YM	A	
*AX-110577792*	4A	674369223	2.3E-05	8.8	653.4–687.1	TKW/SL	A	
*AX-109901470*	4B	15795054	2.8E-05	8.7	15.8–17.3	SL/TKW	G	
*AX-109294476*	4D	13447428	3.1E-05	8.9	13.5	SL	C	
*AX-110185031*	5A	20775669	4.2E-06	10.3	20.7	SL/NSS	G	Li et al. (2019)
*AX-108851118*	5A	519200575	2.7E-05	8.8	517.5–529.3	SL/NS	G	Li et al. (2018)
*AX-109600316*	5A	705150342	3.4E-06	10.4	705.2	SN	G	Gao et al. (2015) and Li et al. (2019)
*AX-110606057*	5B	697524601	7.2E-06	9.9	677.9–697.5	SL	C	Li et al. (2019)
*AX-110510530*	5D	548322186	9.4E-05	7.6	547.6–548.3	YM	C	
*AX-94478216*	6A	3983883	1.7E-06	11.2	2.5–4.0	YM/NSS/SL	T	
*AX-110172686*	6A	538918974	3.9E-05	8.9	538.9–539.3	NS	A	
*AX-109862690*	6A	619409256	2.9E-07	16.7	619.4	YM	A	
*AX-111569068*	6B	132435739	3.3E-05	8.5	132.4–133.0	SL/SN	A	
*AX-94621559*	6D	8684658	6.0E-05	8.1	8.6	SL/SN	T	
*AX-111694627*	6D	312832201	1.4E-05	9.3	312.8	YM	T	
*AX-108976043*	6D	486310967	7.8E-05	7.8	482.3–486.3	NS/YM	T	Li et al. (2019)
*AX-109321162*	7B	585610186	5.1E-05	8.1	585.4–586.4	NS	G	
*AX-111622533*	7D	21913685	5.8E-05	8.1	21.9	SN	G	
*AX-109984815*	7D	142702326	3.5E-05	8.6	138.9–148.9	SN	G	

*^a^The peak marker of the loci.*

*^b^According to the IWGSC v2.1.*

*^c^SNU, spike number per unit area; SN, spikelet number; SL, spike length; KNS, kernel number per spike; TKW, thousand-kernel weight; GY, grain yield.*

Of the loci identified in this study, 28 were identified in the BLUP. Besides this, five loci (249.7–250.3 Mb on 1D, 10.7–21.7 Mb on 3A, 640.8–652.0 Mb on 4A, 516.7–528.5 Mb on 5A, and 460.8–464.8 Mb on 6D) existed in two or more environments and could not be detected in the BLUP. Besides this, four loci (30.2 Mb on 2A, 544.2–544.9 Mb on 5D, 615.2 Mb on 6A, 291.1 Mb on 6D, and 580.8–581.0 Mb on 7B) with strong environmental specificity and only identified in specific environment. Also, we observed a new QTL at 536.4–536.8 Mb on chromosome 6A, which is only identified in BLUP and not observed by analyzing individual environments.

### Candidate Genes

In this study, six candidate genes for GY traits were identified (Table 3). Two cytokinin ribosides (*TraesCS2B02G397600* and *TraesCS3B02G281000*) were identified in the LD decay of the loci on 2B (592.8 Mb) and 3B (480.8–481.4 Mb). Another gene encoding an E3 ubiquitin transferase (*TraesCS3A02G344600*) was identified in the LD decay of the loci on chromosome 3A (596.0 Mb). For the loci on chromosome 3A (696.4–700.9 Mb) and 6A (2.5–4.0 Mb), candidate gene (*TraesCS3A02G459800*) for F-box proteins and serine/threonine-protein kinases (*TraesCS7B02G328800*) were identified, respectively. Besides this, the gene *TraesCS6A02G301800* encoding trehalose 6-phosphatase (T6P) was identified as the candidate gene for the loci 7B (586.4 Mb).

**TABLE 3:** The details for the candidate genes of GY-related traits.

Chromo some	Region (Mb)^a^	Candidate gene^b^	Annotation^c^
2B	592.8	TraesCS2B02G397600	cytokinin riboside
3A	696.4–700.9	TraesCS3A02G344600	E3 ubiquitin transferase
3A	596.0	TraesCS3A02G459800	F-box proteins
3B	480.4–481.4	TraesCS3B02G281000	cytokinin riboside
6A	2.5–4.0	TraesCS6A02G301800	trehalose 6-phosphatase
7B	585.6	TraesCS7B02G328800	serine/threonine-protein kinases

*^a,b,c^According to IWGSC v2.1.*

## Discussion

The 251 spring wheat accessions were divided into three subgroups (Figure 1), and the characterization of the subgroups was largely consistent with geographic origins, released years, and pedigrees. For example, most of the accessions from Heilongjiang province ranging from the 1950s to 1980s belonged to subgroup 1, the accessions from Heilongjiang province ranging from the 1990s to 2010s belonged to subgroup 2, and subgroup 3 mainly included the accessions from the Jilin province and foreign counties. A significant population structure existed in the diverse panel, and previous studies indicate that the lack of appropriate correction for population structure can lead to spurious MTAs (Zhu et al., 2008). Thus, to eliminate spurious MTAs, an MLM model with subpopulation data (Q) (fixed-effect factors) and kinship matrix (random-effect factor) were conducted. Also, the LD decay influenced several factors, such as population structure, allele frequency, recombination rate, and selection, and seriously affects the precision of association mapping. In this panel, the LD decay for the whole genome was about 8 Mb, consistent with previous reports (Liu et al., 2017), and indicates that the number of markers is enough for the subsequent association analysis.

### Comparison With the QTL or Gene in Previous Studies

The genes or loci associated with GY-related traits were extensively reported previously. In this study, association of GY and related traits were performed based on the wheat high-density physical map. GY is a typical quantitative inheritance complex trait and significantly influenced by various environments (Gao et al., 2015; Li et al., 2018). The GY-related loci (gene) is distributed on all 21 chromosomes in wheat (Kumar et al., 2007; Reif et al., 2011; Lee et al., 2014; Lopes et al., 2015; Sukumaran et al., 2015; Gao et al., 2017; Li et al., 2018). Azadi et al. (2015) report a QTL for GY-related traits on chromosome 1A, which is tightly linked with the SSR marker *gwm357* and located between the two GY QTL mapped by Cuthbert et al. (2008) and Huang et al. (2004). Also, a locus for KNS at chromosome 1A (around 26.8–40.5 Mb) is identified (Wang et al., 2011; Li et al., 2019). According to the IWGSC V2.0 and the consensus map by Maccaferri et al. (2015), the loci in 1A identified by Azadi et al. (2015) and Li et al. (2019) are overlapped with the 1A locus (28.6–30.7 Mb) for TKW and SL identified in the present study. Thus, these loci in 1A (*AX-108817901* 307.1–321.1 Mb) for TKW or SL (*AX-109971512* 338.9–346.0 Mb) for SL (*AX-108850659* 370.7–370.8 Mb) for SL and (*AX-110363533* 587.8–594.7 Mb) for SN were novel. Besides this, the loci for YM and NS identified in the 1B chromosome (*AX-110953049* 100.8–103.2 Mb) and two loci in 1D chromosome for YM and NS (*AX-110953049* 252.4–284.1 Mb) and SL (*AX-94767476* 430.1–430.3 Mb) may be novel.

Li et al. (2019) identified a locus for GY at chromosome 2A (33.3–34.9 Mb) and 3A (21.2–26.9 and 702.6–712.2 Mb) by association mapping in 166 wheat accessions. Also, we identified few loci for GY-related traits in chromosome 2A and 3A. Of these loci identified by Li et al. (2019), the locus at 2A (32.3–33.9 Mb) was overlapped with the QTL for GY at 2A (34.7 Mb) in this study; the locus at 3A (22.9–39.3 Mb) were nearly with the QTL for GY at 3A (10.9–21.9 Mb), whereas the locus at 3A (700.1–705.1 Mb) coincides with the locus for KNS on 3A (696.4–700.9 Mb) in our study. Besides this, Azadi et al. (2015) and Gao et al. (2015) identified a locus at the 3A chromosome nearly the 3A locus (696.4–700.9 Mb) identified in this study. On chromosome 3D, Li et al. (2019) identified a locus for KNS by GWAS in 166 common wheat accessions (574.8–576.8 Mb) and explained 7.1–9.9% of the phenotypic variations, which overlapped with the loci for NS and SL (3D, 600.0–600.4 Mb) identified in our study. The loci on chromosome 2B (*AX-111470278* 592.8 Mb) for SN and YM, 2D (*AX-86163393* 23.9–37.9 Mb) for SL and NSS, and *AX-110090611* (556.2–556.3 Mb) for SL are likely to be new loci for GY-related traits. Besides this, the loci identified in chromosome 3A (*AX-110922897* 3A 596.0 Mb) for NSS and SL, 3B (*AX-109379472* 480.4–481.4 Mb) for NSS, *AX-108791993* (589.9 Mb) for SL and 3D (*AX-94879852* 22.8 Mb) for NS and YM appear to be new. Also, the loci identified in chromosome 4A (*AX-111662342* 4A 640.2 Mb) for YM, *AX-110577792* (653.4–687.1 Mb) for TKW and SL, 4B (*AX-109901470* 15.8–17.3 Mb) for SL and TKW, and *4D* (*AX-109294476* 13.5 Mb) are new loci for GY-related traits.

The 5A chromosome enriched a series of loci for GY-related traits. Reif et al. (2011) identified a GY QTL on 5A linked with SSR marker *barc151*. Liu et al. (2014) also mapped a SL QTL on 5A. Li et al. (2018) identified a QTL *QSL.caas-5AL.2* for SL in the Gaocheng 8901 × Zhou 8425B population. Besides this, Li et al. (2019) identified a locus for TKW by GWAS in 166 common wheat accessions at 5A (709.4–711.3) and explained phenotypic variations 8.2–13.0%. The locus for SL identified in this study at chromosome 5A (705.2 Mb) is overlapped with the loci discussed above. Azadi et al. (2015) detected a QTL for KNS on 5B, linked with DArT markers *wPt-3661*, whereas the 5B QTL is around the loci for SL (677.9–697.5 Mb) identified in the present study, and these might be the same. Also, the SL locus (677.9–697.5 *Mb*) on 5B is nearly the loci for KL, KW, and TKW detected by Chen et al. (2016); Mohler et al. (2016), and Sun et al. (2017), respectively, indicating that this should be an important locus in determining kernel weight. Li et al. (2019) identified a locus for TKW and SN by GWAS in 166 wheat accessions at 5B (696.5–699.7 Mb) and explained 8.2–13.0% of the phenotypic variations, which are nearly the loci at chromosome 5B identified in our study. Also, Li et al. (2019) detected a locus for TKW and SN by GWAS at 6D (482.3–486.3 Mb). We also have identified a locus for NS and GY on chromosome 6D (482.3–486.3 Mb). Thus, the loci on chromosome 5D (*AX-110510530* 547.6–548.3 Mb) for YM and 6A (*AX-94478216* 2.5–4.0 Mb) for YM, NSS, and SL and the loci (*AX-110172686* 538.9–539.3 Mb) for NS and (*AX-109862690* 619.4 Mb) for YM are likely to be new. The loci for GY-related traits identified in chromosome 6B (*AX-111569068* 132.4–133.0 Mb) for SL and SN, 6D (*AX-94621559* 8.6 Mb) for SL and SN, and *AX-111694627* (312.8 Mb) for YM were novel. Besides this, we identified 3 loci on the 7B and 7D chromosomes for GY-related traits. The loci on chromosome 7B (*AX-109321162* 585.4–586.4 Mb) for NS and 7D (*AX-111622533* 21.9 Mb; *AX-109984815* 138.9–148.9 Mb) for SN appear to be new.

Among the 38 loci for GY and related traits, 10 loci discussed above (1A: 28.6–30.7 Mb, 2A: 30.2 34.7 Mb, 3A: 10.9–21.9 Mb, 3A: 696.4–700.9 Mb, 3D: 600.0–600.4 Mb, 5A: 10.3–20.7 Mb, 5A: 517.5–529.3 Mb, 5A: 702.6–705.2 Mb, 5B: 677.9–697.5 Mb, and 6D: 482.3–486.3 Mb) should be the same as the QTL reported in previous studies. The stable loci validated by both GWAS and QTL mapping between ours and previous studies indicate that they are widespread in varieties and may be more powerful and stable in various varieties. Moreover, the methods of GWAS used in the present study are proven to be reliable and efficient in detecting loci for GY and related traits.

Different QTL should be used in different regions. According to the results from association analysis, most of the loci (28) identified in this study existed on two or more environments (including BLUP), indicating high stability, and could be applied in various regions; these loci are only identified in an individual environment (30.2 Mb on 2A, 544.2–544.9 Mb on 5D, 615.2 Mb on 6A, 291.1 Mb on 6D, and 580.8–581.0 Mb on 7B) could be used in a specific environment, whereas those loci (536.4–536.8 Mb on chromosome 6A) only detected in BLUP may provide new insights into the genetic mechanism of GY-related traits.

### Candidate Gene Analysis

To identify the candidate genes for GY-related traits, the flanking sequences of SNP markers (including the markers from the LD decay interval) significantly associated with GY-related traits were used as queries to BLAST against the National Center for Biotechnology Information (NCBI) and European Nucleotide Archive (ENA) databases. In total, six candidate genes were identified (Table 3) for further research. Of these, two cytokinin ribosides (*TraesCS2B02G397600* and *TraesCS3B02G281000*) were identified in the LD decay of the loci on chromosome 2B (592.8 Mb) and 3B (480.4–481.4 Mb). The cytokinin is a positive regulator of shoot growth and negative regulator of root growth (Han et al., 2014) and has been strongly implicated in many aspects affecting GY-related traits, particularly the kernel number and size (Yamburenko et al., 2017). Besides this, cytokinin also plays crucial roles in the response to biotic and abiotic stressors (Cortleven et al., 2019). *TraesCS3A02G344600*, coded an E3 ubiquitin transferase, which were identified for the loci on chromosome 3A (596.0 Mb). Previous studies report that the E3-Ubiquitin protein ligases are a large protein family that is important in plant growth and development (Craig et al., 2009; Park et al., 2010). Wang et al. (2020) indicates that a E3 ligase gene, *TaSDIR1-4A*, contributes to the determination of grain size in common wheat. For the loci on chromosome 3A (696.4–700.9 Mb) and 6A (2.5–4.0 Mb), candidate genes for F-box proteins (*TraesCS3A02G459800*) and serine/threonine-protein kinases (*TraesCS7B02G328800*) were identified, respectively. F-box proteins are a large protein family and plays crucial roles in cell-cycle progression, transcriptional regulation, flower formation, signal transduction, and many other cellular processes in plants (Hong et al., 2012; Kaur et al., 2017; Marcotuli et al., 2018; Guérin et al., 2021). Furthermore, *AX-108951749* on 2B and *IWA2223* on 5AL encodes the serine/threonine-protein kinases. The serine/threonine-protein kinases play crucial roles in cell-cycle progression, transcriptional regulation, flower formation, and signal transduction (Rehman et al., 2019; Jia et al., 2020). The gene *TraesCS6A02G301800* encodes trehalose 6-phosphatase (T6P) on the loci 7B (585.6 Mb), which regulates carbon assimilation and sugar status in plants. In addition, previous studies report that T6P has also been demonstrated to play an essential role in plant development under abiotic stress (Diab et al., 2013; Gahlaut et al., 2019).

### Potential Implications in Wheat Breeding

The conventional breeding approach has led to improved GY on wheat, and breeding selection is time-consuming and not very efficient (He et al., 2010). Significant additive effects are reported between GY traits and a number of favorable alleles, indicating that pyramiding favorable alleles will improve GY traits (Li et al., 2018). The markers associated with GY detected should facilitate MAS. Loci with pleiotropic and consistent effects across each environment should be amenable to MAS. Besides this, the loci validated between ours and previous studies by QTL-mapping or GWAS indicated these loci are stable in various varieties and should be applied in further study. In this study, accessions with agronomic characters and more favorable alleles, such as Kefeng 6, Beimai 15, Kehan 18, Longfumai 6, Longfumai 196, Kechun 7, Longmai 23, Kechun 1, Longfumai 4, and Longmai 13, are recommended as parental lines for improvement of GY-related traits in wheat breeding.

## Conclusion

In this study, association mapping for GY and related traits (SNU, SL, SN, KNS, and TKW) were conducted in a diverse panel, including 251 spring wheat varieties mainly from China. In total, 38 loci were identified, and each explained 6.5–16.7% of the phenotypic variations. Of these, 12 are overlapped with known genes or QTL, and 26 are likely to be new. Besides this, six candidate genes for GY-related traits involved in grain development, plant hormone signal transduction, and starch biosynthesis were identified. The stable loci and associated markers and varieties with favorable traits and alleles could be used in further wheat breeding. These new loci provide a new sight in genetic architecture of GY-related traits.

## Data Availability Statement

The original contributions presented in the study are included in the article/Supplementary Material, further inquiries can be directed to the corresponding author/s.

## Author Contributions

YL and LY designed the research. JT analyzed the physiology data. YL and WY drafted the manuscript. YL, YS, JC, and CT performed the experiment. HZ and LY revised the manuscript. All authors have read, edited, and approved the current version of the manuscript.

## Conflict of Interest

The authors declare that the research was conducted in the absence of any commercial or financial relationships that could be construed as a potential conflict of interest.

## Publisher’s Note

All claims expressed in this article are solely those of the authors and do not necessarily represent those of their affiliated organizations, or those of the publisher, the editors and the reviewers. Any product that may be evaluated in this article, or claim that may be made by its manufacturer, is not guaranteed or endorsed by the publisher.

## Footnotes

1
http://www.sas.com

2
https://urgi.versailles.inra.fr/blast_iwgsc/blast.php

3
http://pritchardlab.stanford.edu/structure.html

4
http://www.ncbi.nlm.nih.gov/

## Abbreviations

BLUE: best linear unbiased estimation
GY: grain yield
GWAS: genome-wide association study
*h* ^2^: broad-sense heritability
KASP: kompetitive allele-specific PCR
KNS: kernel number per spike
LD: linkage disequilibrium
MAS: marker-assisted selection
QTL: quantitative trait loci
*R* ^2^: phenotypic variance explained
RIL: recombinant inbred line
SL: spike length
SN: spike number per unit area
SNP: single nucleotide polymorphism
TKW: thousand-kernel weight.
