# Supplementary material for: The Genetic Architecture of Grain Yield in Spring Wheat Based on Genome-Wide Association Study
Source: Front Genet. 2021 Nov 15;12:728472. doi: 10.3389/fgene.2021.728472 (PMC8634730; doi:10.3389/fgene.2021.728472)
Supplement: Supplementary file 1 [file Data_Sheet_1.zip › Supplementary material/Figure S1.docx]

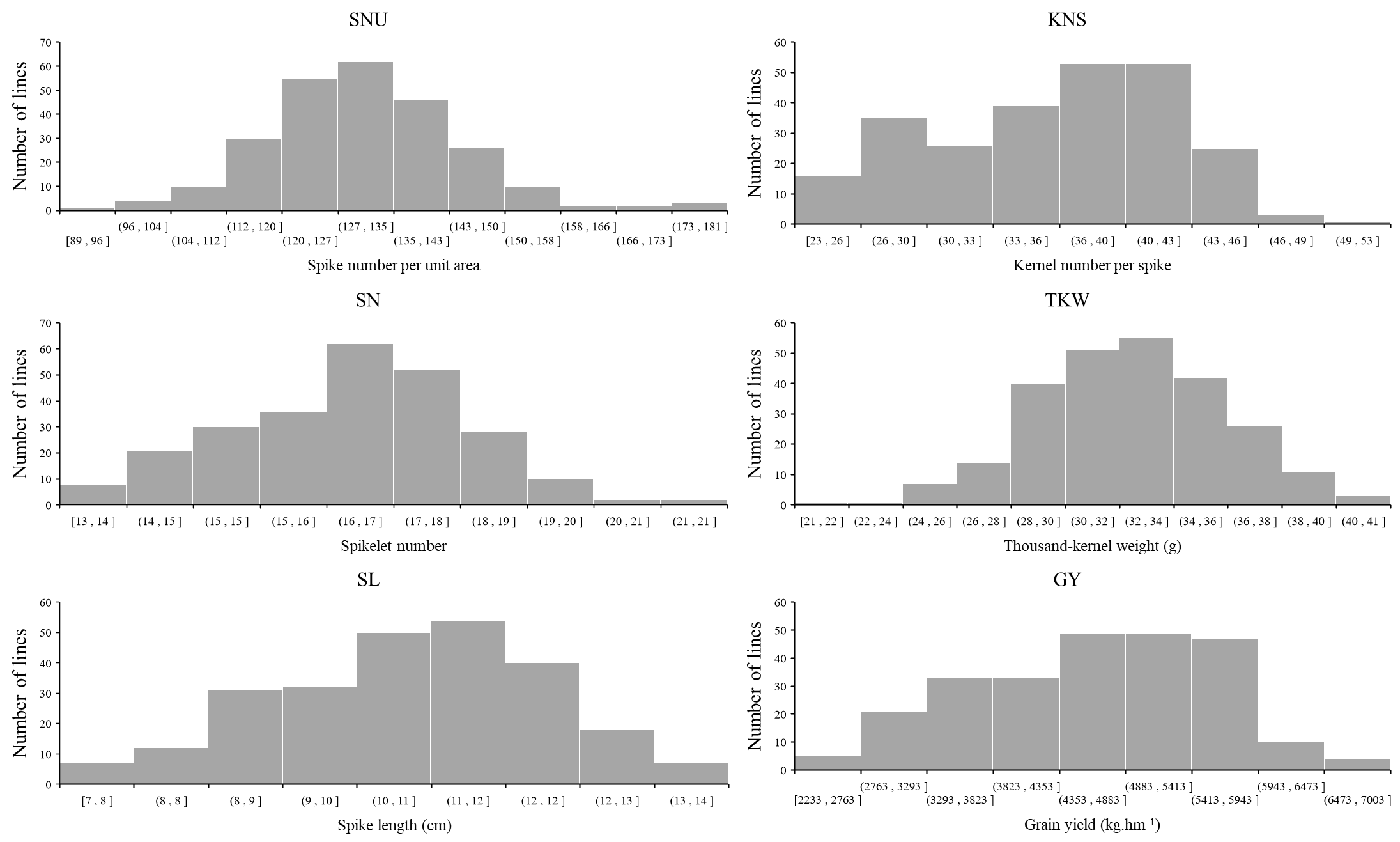


**Fig. S1** Frequency distributions for grain related traits (best linear unbiased prediction (BLUP)) of 251 wheat accessions.

SNU: spike number per unit area; SN: spikelet number; SL: spike length; KNS: kernel number per spike; TKW: thousand-kernel weight; GY: grain yield.

WGSC (http://www.wheatgenome.org/)
