# Supplementary material for: The Genetic Architecture of Grain Yield in Spring Wheat Based on Genome-Wide Association Study
Source: Front Genet. 2021 Nov 15;12:728472. doi: 10.3389/fgene.2021.728472 (PMC8634730; doi:10.3389/fgene.2021.728472)
Supplement: Supplementary file 1 [file Data_Sheet_1.zip › Supplementary material/Figure S2.docx]

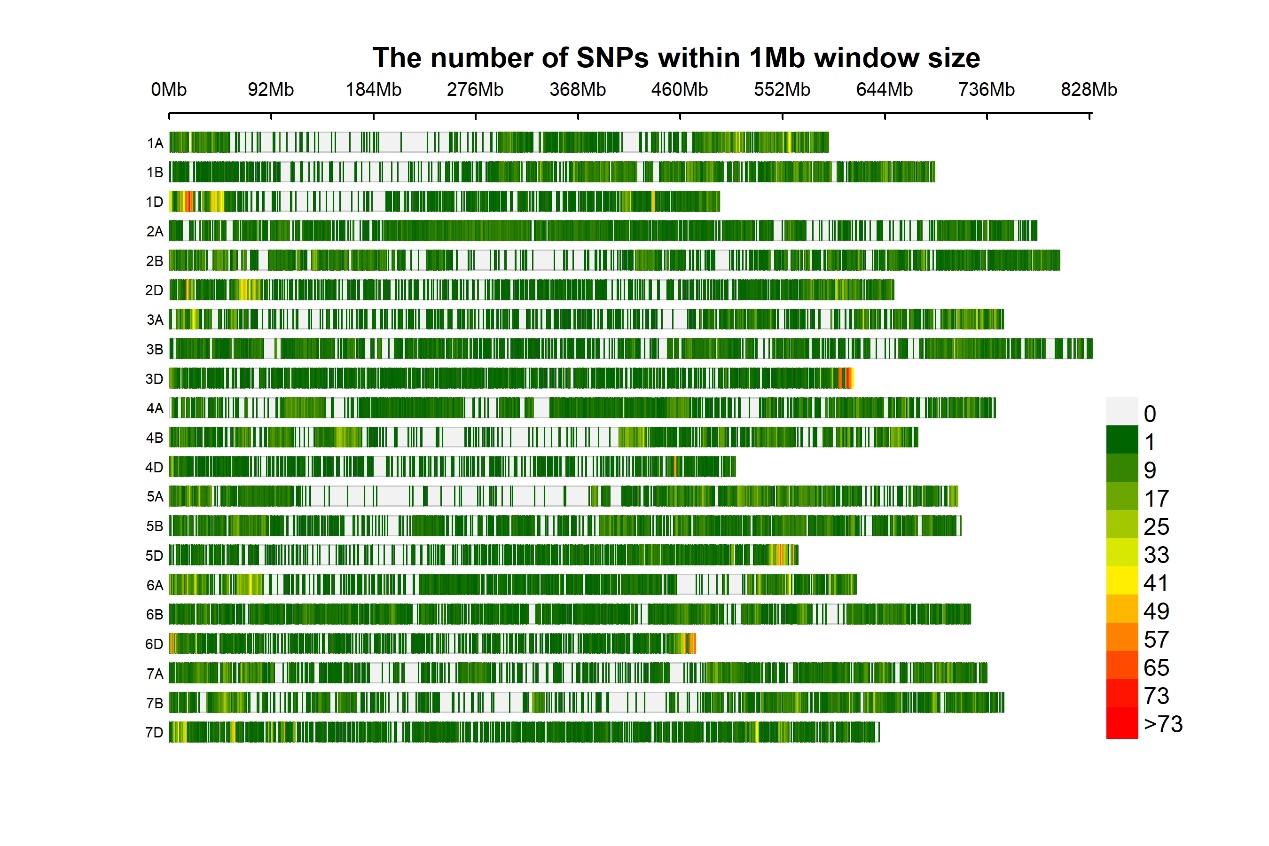


**Fig. S2** Marker density on each chromosome of the GWAS panel genotyped using the wheat 55 K SNP array. Different colors represent the corresponding number of SNPs within a 1 Mb distance on each of the 21 chromosomes (based on IWGSC 2.1).
