# Supplementary material for: The Genetic Architecture of Grain Yield in Spring Wheat Based on Genome-Wide Association Study
Source: Front Genet. 2021 Nov 15;12:728472. doi: 10.3389/fgene.2021.728472 (PMC8634730; doi:10.3389/fgene.2021.728472)
Supplement: Supplementary file 1 [file Data_Sheet_1.zip › Supplementary material/Figure S4.docx]

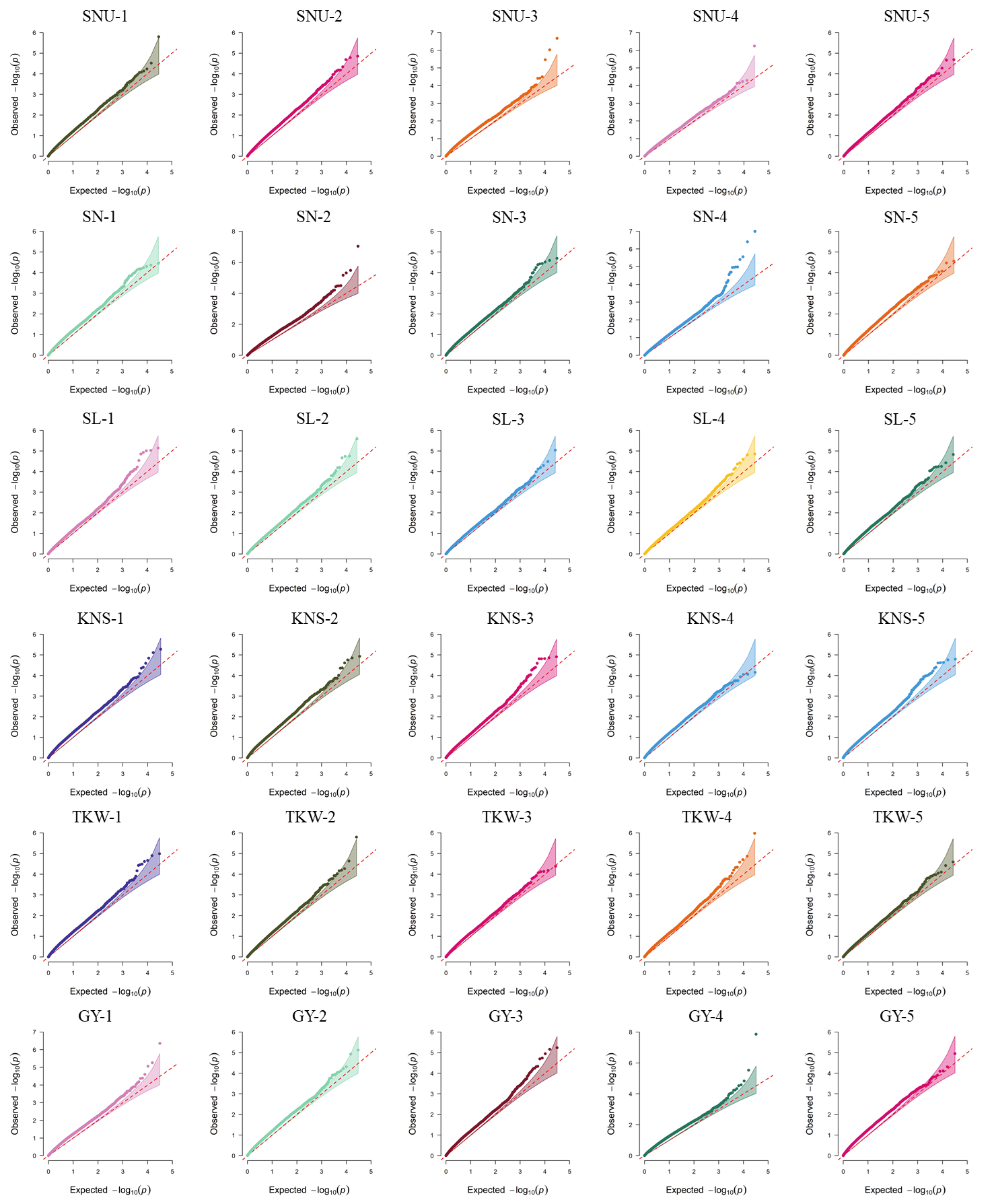


**Fig. S4** Quantile-quantile (Q-Q) plot for grain yield related traits in 251 wheat accessions analyzed by the mixed linear model (MLM) in Tassel v5.0.

SNU: spike number per unit area; SN: spikelet number; SL: spike length; KNS: kernel number per spike; TKW: thousand-kernel weight; GY: grain yield.

The 1, 2, 3, 4 and 5 indicated the Haerbin 2018; Haerbin 2019, Keshan 2018, Keshan 2019 and the best linear unbiased prediction (BLUP).
