# Supplementary material for: The Genetic Architecture of Grain Yield in Spring Wheat Based on Genome-Wide Association Study
Source: Front Genet. 2021 Nov 15;12:728472. doi: 10.3389/fgene.2021.728472 (PMC8634730; doi:10.3389/fgene.2021.728472)
Supplement: Supplementary file 1 [file Data_Sheet_1.zip › Supplementary material/Table S2.docx]

**Table S2** Pairwise correlation coefficients among grain yield related traits in 251 spring wheat accessions

| Traits | SNU | SN | SL | KNS | TKW | GY |
| --- | --- | --- | --- | --- | --- | --- |
| SNU | 1.00 |  |  |  |  |  |
| SN | -0.24* | 1.00** |  |  |  |  |
| SL | -0.24* | 0.81** | 1.00** |  |  |  |
| KNS | -0.24* | 0.83** | 0.72** | 1.00** |  |  |
| TKW | -0.20* | 0.11* | 0.24** | 0.20* | 1.00** |  |
| GY | 0.18* | 0.57** | 0.57** | 0.76** | 0.56** | 1.00** |

* significant at *P*<0.05; ** significant at *P*<0.01.

SNU: spike number per unit area; SN: spikelet number; SL: spike length; KNS: kernel number per spike; TKW: thousand-kernel weight; GY: grain yield.
