# Supplementary material for: The Genetic Architecture of Grain Yield in Spring Wheat Based on Genome-Wide Association Study
Source: Front Genet. 2021 Nov 15;12:728472. doi: 10.3389/fgene.2021.728472 (PMC8634730; doi:10.3389/fgene.2021.728472)
Supplement: Supplementary file 1 [file Data_Sheet_1.zip › Supplementary material/Table S3.docx]

Table S3 Basic statistical analysis of SNP markers used in GWAS for 251 spring wheat accessions

| Chromosome | No. of markers | Distance (Mb) ^a^ | Density (Mb/marker) |  | MAF | |  |
| --- | --- | --- | --- | --- | --- | --- | --- |
|  |  |  |  |  | Mean | Range |  |
| 1A | 2676 | 593.3 | 0.222 |  | 0.24 | 0.05-0.50 |  |
| 1B | 2672 | 688.7 | 0.258 |  | 0.30 | 0.05-0.50 |  |
| 1D | 2585 | 495.2 | 0.192 |  | 0.19 | 0.05-0.48 |  |
| 2A | 2679 | 780.8 | 0.291 |  | 0.25 | 0.05-0.50 |  |
| 2B | 2680 | 801.2 | 0.299 |  | 0.25 | 0.05-0.50 |  |
| 2D | 2671 | 651.7 | 0.244 |  | 0.28 | 0.05-0.47 |  |
| 3A | 2241 | 750.6 | 0.335 |  | 0.25 | 0.05-0.50 |  |
| 3B | 2672 | 830.3 | 0.311 |  | 0.24 | 0.05-0.50 |  |
| 3D | 2135 | 615.5 | 0.288 |  | 0.20 | 0.05-0.48 |  |
| 4A | 2675 | 743.3 | 0.278 |  | 0.25 | 0.05-0.50 |  |
| 4B | 2652 | 673.5 | 0.254 |  | 0.22 | 0.05-0.49 |  |
| 4D | 1118 | 509.5 | 0.456 |  | 0.26 | 0.05-0.48 |  |
| 5A | 2686 | 709.2 | 0.264 |  | 0.25 | 0.05-0.50 |  |
| 5B | 2682 | 712.4 | 0.266 |  | 0.28 | 0.05-0.50 |  |
| 5D | 2203 | 565.7 | 0.257 |  | 0.25 | 0.05-0.50 |  |
| 6A | 2682 | 618.0 | 0.230 |  | 0.25 | 0.05-0.48 |  |
| 6B | 2691 | 721.0 | 0.268 |  | 0.26 | 0.05-0.50 |  |
| 6D | 2125 | 473.5 | 0.223 |  | 0.25 | 0.05-0.50 |  |
| 7A | 2680 | 735.5 | 0.274 |  | 0.26 | 0.05-0.50 |  |
| 7B | 2619 | 750.6 | 0.287 |  | 0.25 | 0.05-0.50 |  |
| 7D | 2679 | 638.6 | 0.238 |  | 0.22 | 0.05-0.50 |  |
| A genome | 18319 | 4930.7 | 0.271 |  | 0.25 | 0.05-0.50 |  |
| B genome | 18668 | 5177.6 | 0.277 |  | 0.26 | 0.05-0.50 |  |
| D genome | 15516 | 3949.8 | 0.271 |  | 0.24 | 0.05-0.48 |  |
| Whole | 52503 | 14058.1 | 0.273 |  |  |  |  |

^a^ The physical map based on wheat genome sequences from the IWGSC (http://www.wheatgenome.org/)
