# Supplementary figures and images for: The Genetic Architecture of Grain Yield in Spring Wheat Based on Genome-Wide Association Study
Source: Front Genet. 2021 Nov 15;12:728472. doi: 10.3389/fgene.2021.728472 (PMC8634730; doi:10.3389/fgene.2021.728472)

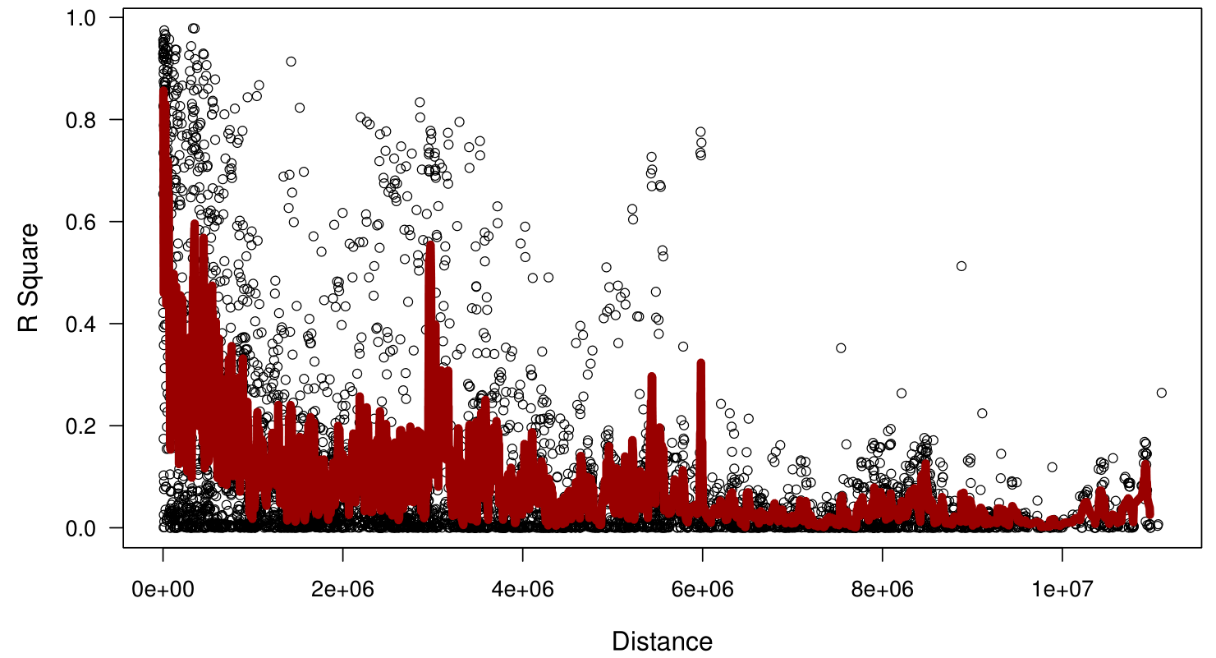


**Fig. S3** The LD decay for all the whole genome of the 251 accessions

Supplement: Supplementary file 1 [file Data_Sheet_1.zip › Supplementary material/Figure S3.docx]
